# Significant Impact of Sequence Variations in the Nucleoprotein on CD8 T Cell-Mediated Cross-Protection against Influenza A Virus Infections

**DOI:** 10.1371/journal.pone.0010583

**Published:** 2010-05-11

**Authors:** Weimin Zhong, Feng Liu, Libo Dong, Xiuhua Lu, Kathy Hancock, Ellis L. Reinherz, Jacqueline M. Katz, Suryaprakash Sambhara

**Affiliations:** 1 Influenza Division, National Center for Immunization and Respiratory Diseases, Centers for Disease Control and Prevention, Atlanta, Georgia, United States of America; 2 Department of Medical Oncology, Dana-Farber Cancer Institute, Department of Medicine, Harvard Medical School, Boston, Massachusetts, United States of America; Washington University, United States of America

## Abstract

**Background:**

Memory CD8 T cells to influenza A viruses are widely detectable in healthy human subjects and broadly cross-reactive for serologically distinct influenza A virus subtypes. However, it is not clear to what extent such pre-existing cellular immunity can provide cross-subtype protection against novel emerging influenza A viruses.

**Methodology/Principal Findings:**

We show in the mouse model that naturally occurring sequence variations of the conserved nucleoprotein of the virus significantly impact cross-protection against lethal disease *in vivo*. When priming and challenge viruses shared identical sequences of the immunodominant, protective NP_366_/D^b^ epitope, strong cross-subtype protection was observed. However, when they did not share complete sequence identity in this epitope, cross-protection was considerably reduced. Contributions of virus-specific antibodies appeared to be minimal under these circumstances. Detailed analysis revealed that the magnitude of the memory CD8 T cell response triggered by the NP_366_/D^b^ variants was significantly lower than those triggered by the homologous NP_366_/D^b^ ligand. It appears that strict specificity of a dominant public TCR to the original NP_366_/D^b^ ligand may limit the expansion of cross-reactive memory CD8 T cells to the NP_366_/D^b^ variants.

**Conclusions/Significance:**

Pre-existing CD8 T cell immunity may provide substantial cross-protection against heterosubtypic influenza A viruses, provided that the priming and the subsequent challenge viruses share the identical sequences of the immunodominant, protective CTL epitopes.

## Introduction

Infection with one subtype of influenza A virus often results in a strong protection against subsequent infections with heterosubtypic influenza A viruses in animal models. This type of immunity, termed heterosubtypic immunity [1], is unable to prevent infection *per se*, but can considerably reduce viral load, leading to an accelerated recovery from influenza illness [2]. Heterosubtypic immunity is a necessary component of a so-called “universal” influenza vaccine that would provide protection against illness from multiple subtypes of influenza A viruses.

Optimal heterosubtypic immunity is thought to be dependent on multiple components of the immune system. This includes T cells, especially the CD8 T cell subset that recognizes CTL epitopes derived from the conserved internal proteins of the virus such as nucleoprotein (NP) and matrix protein 1 (M1) [2], haemagglutinin (HA)-specific mucosal IgA antibodies [3], and serum antibodies specific for the ecotodomain of the highly conserved matrix protein 2 (M2) [4], and possibly, HA-specific antibodies that recognize conserved B cell epitopes of the molecule [5], [6]. Extensive studies in the mouse model have demonstrated that one or other component of the immune system may play a dominant role under different circumstances. For example, many studies have shown that CD8 T cells are the major mediator of heterosubtypic immunity following intranasal priming of immune competent mice with live influenza virus particles [7]–[9]. On the other hand, heterosubtypic immunity was not observed in B cell-deficient mice, although the same challenge route was used and cross-reactive CTLs were detectable in these animals [10].

Seasonal influenza A H1N1 and H3N2 viruses have been circulating in humans for many years [11]. As a result, peripheral blood memory T cells that are broadly cross-reactive, not only against serologically distinct seasonal influenza A viruses but also avian H5N1 influenza viruses, can be demonstrated in healthy individuals [12]–[15]. As a majority of influenza virus-specific memory CD8 T cells are directed against the conserved NP and M1 protein [16], it has long been proposed that pre-existing memory CD8 T cells should, in principal, provide some degree of cross-protection against disease during the emergence of a new pandemic influenza A virus in humans. However, although CD8 T cell mediated heterosubtypic protection is relatively efficient in animal models, such immune benefit remains unclear in humans. Recent analysis of the historic epidemiological data suggests that prior exposure to H1N1 influenza virus resulted in generation of protective immunity against heterosubtypic H2N2 virus [17]. A variety of NP and/or M1-based novel vaccination strategies are currently under evaluation for their efficiency to induce T cell immunity-based protection against heterosubtypic influenza A viruses [18], [19]. However, even the more conserved internal proteins of influenza A viruses, including the NP, undergo evolutionary change [20], [21]. Consequently, multiple CD8 CTL epitope variants have been identified in circulating seasonal influenza A viruses [22], [23]. *In vitro* studies have showed that certain human CD8 CTL clones generated were able to recognize CTL epitope variants derived from both homo- and heterosubtypic influenza A viruses [24]. The impact of such sequence variations on CD8 T cell-mediated heterosubtypic immunity *in vivo* has not been examined previously.

In the present study, we assessed the ability of cross-reactive memory CD8 T cells to the immunodominant, protective NP_366_/D^b^ CTL epitope to confer protective heterosubtypic immunity under circumstance where priming and subsequent challenge influenza A viruses do or do not share sequence identity of the CTL epitope. Our results reveal that sequence variations in the NP of influenza A viruses can significantly impact heterosubtypic protection mediated by the NP_366_/D^b^-specific memory CD8 T cells *in vivo*, under circumstances in which virus-specific serum HI antibodies, nasal IgA and serum M2-specific antibodies were unlikely to impact on the protective heterosubtypic immunity.

## Results and Discussion

In the present study, we chose the C57BL/6 (B6) mouse model to assess the extent of heterosubtypic immunity based on the following considerations: (1) Both CD8 and CD4 T cell epitope repertoire of influenza A virus have been extensively characterized in B6 mice and a D^b^-restricted, immunodominant NP_366_ epitope is the major target to mediate a protective, memory CTL response after secondary infections with influenza A viruses [25]–[28]. (2) Analysis of the deduced amino acid sequences of the NP gene from a large number of influenza A viruses (955 sequences), indicated that, certain amino acid residues within the NP_366_/D^b^ CTL epitope undergo constant evolutionary change (Table 1), despite the overall conserved nature of this internal protein between different influenza A virus subtypes (∼89% identity among sequences analyzed). A total of ten naturally occurring NP_366_ variants were identified, each representing a different subtype of influenza A virus. Of interest, all of the amino acid substitutions observed were located at the C-terminal bulge of the NP_366_ peptide backbone, the featured structural region of the NP_366_/D^b^ complex exposed for recognition by the TCRs of CD8 T cell subset induced by influenza A virus infection [29]. (3) Except for the NP_366_/D^b^ epitope, each panel of heterosubtypic influenza A viruses used for priming and secondary challenge in the present study share identical immunodominant MHC class II T cell epitopes and all other known class I T cell epitopes (Table S1 and Table S2). Such a combination of priming and challenge viruses offers an unique opportunity to dissect the impact of NP_366_/D^b^ epitope sequence variation on memory CD8 T cell-mediated protective heterotypic immunity in B6 mice.

**Table 1:** Naturally occurring NP_366_/D^b^ CTL epitope variants of influenza A viruses^1^

Influenza A virus strain	Subtype	Peptide name	Peptide sequence
A/Puerto Rico/8/1934	H1N1	NP_366_WT	A S N E N M E T M
A/Taiwan/01/1986	H1N1	NP_366_E-D	A S N E N M D T M
A/Japan/305/1957	H2N2	NP_366_E-D	A S N E N M D T M
X31	H3N2	NP_366_WT	A S N E N M E T M
A/NT/60/1968	H3N2	NP_366_ET-DA	A S N E N M D A M
A/Hong Kong/127/1982	H3N2	NP_366_M-V	A S N E N V E T M
A/Memphis/102/1972	H3N2	NP_366_E-D	A S N E N M D T M
A/Memphis/6/1990	H3N2	NP_366_ET-DN	A S N E N M D N M
A/Hong Kong/156/1997	H5N1	NP_366_MT-VA	A S N E N V E A M
A/Vietnam/1203/2004	H5N1	NP_366_T-A	A S N E N M E A M
A/duck/Guangxi/1793/2004	H5N1	NP_366_M-L	A S N E N L E T M
A/chicken/Guiyang/3570/2005	H5N1	NP_366_M-I	A S N E N I E T M
A/chicken/Korea/S1/2003	H9N2	NP_366_M-T	A S N E N T E T M

1 A pool of 995 amino acid sequences of influenza A virus nucleoprotein were retrieved from the Entrez protein database of National Center for Biotechnology Information (NCBI). This includes 46 NP sequences isolated from wild birds (quail and gull), 546 from domestic birds (chicken, duck and goose), 187 from humans, 156 from swine, and 20 from equine. Each NP sequence was derived from one isolate of influenza A virus. Sequence alignment was performed by using MAFF (version 5.8) multiple sequence alignment program accessible at http://us.expasy.org. Listed are the ten naturally occurring D^b^-restricted NP_366_ variants with amino acid mutations at the potential TCR contact positions (position 4, 6, 7 and 8 of the peptides). Mutations at the primary and secondary D^b^ anchor positions of the NP366 variants (position 3, 5 and 9) are anticipated to result in the considerable loss of the peptide binding to the MHC molecule, thus not included for further experimental analyses in the present study. One representative strain of influenza A virus for each NP_366_ variant identified is listed to illustrate the serological heterogeneity of the influenza A viruses that bear these CTL epitope mutations in nature. Amino acids underlined represent mutations relative to PR8-NP_366_ sequence.

### Heterosubtypic protection is significantly decreased when challenge influenza A viruses do not share identical NP_366_/D^b^ CTL epitopes as the priming virus

**Figure 1:**
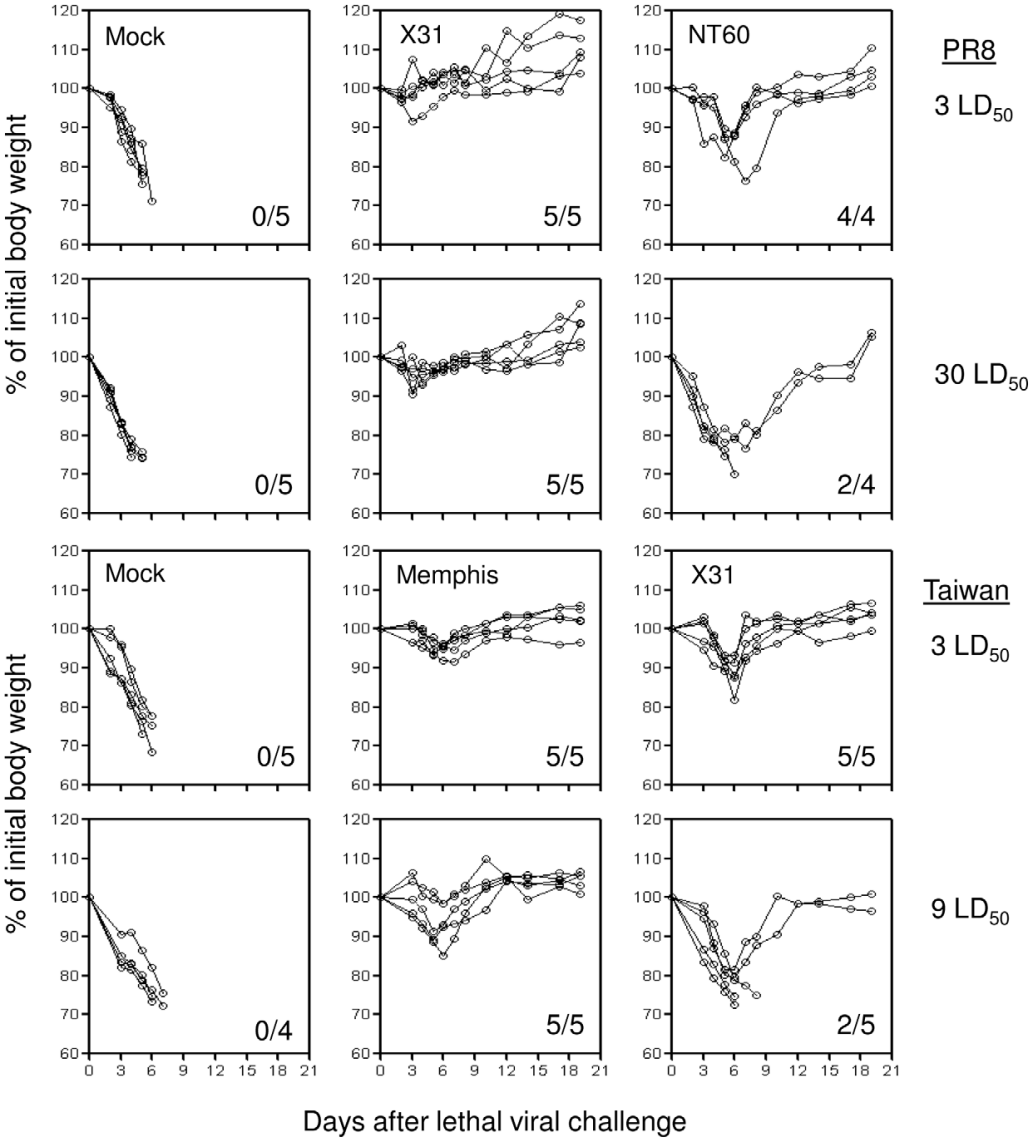
Body weight loss and survival after heterosubtypic influenza virus infections. Five to eight B6 mice per group were primed intranasally with X31 or Memphis H3N2 influenza A viruses as indicated or equal volume of allantoic fluid as control. 35 to 45 days after priming, the animals were challenged with lethal doses of heterosubtypic H1N1 influenza viruses as indicated. Body weight loss (left panel) and survival (right panel) of the animals were monitored until day 19 after lethal challenge. Animals that lost 25% of their initial body weight were considered moribund and sacrificed according the animal protocol. The results were expressed as body weight loss of individual mice per group. The numbers on the lower right corner of each graph indicate survival rate of each group (the number of animals survived/total number of animals tested).

We first evaluated the extent of heterosubtypic protection against lethal challenge with viruses that do or do not share the identical NP_366_/D^b^ CTL epitope as the priming viruses. As expected from previous studies [7], [8], mice that were primed by intranasal infection with X31 virus (H3N2) did not show any body weight loss following intranasal challenge with a low lethal dose (3 LD_50_) of heterosubtypic PR8 virus (H1N1) that shares complete sequence identity in the NP_366_ CTL epitope with X31 virus (Fig. 1, upper panel). To further evaluate the robustness of the heterosubtypic protection induced under this circumstance, a second group of X31-primed mice received a 10-fold higher lethal challenge dose (30 LD_50_) of PR8 virus. Even in this case, X31-primed mice exhibited only modest and transient weight loss on day five after the lethal viral challenge (maximum mean weight loss: ∼5%) and rapidly regained the weight to normal levels by day seven post-challenge. All X31-primed animals survived challenge with either lethal dose of PR8 virus. In contrast, mice that were primed by NT60 virus (H3N2) and then challenged with 3 LD_50_ of PR8 virus, lost approximately ∼10% of body weight between day 7 and 9 post-challenge. When a second group of NT60-primed mice were challenged with high lethal dose (30 LD_50_) of PR8 virus, mice experienced severe weight loss. Only 50% of the mice survived in this group of animals. Control mice challenged with either doses of PR8 virus experienced substantial and rapid weight loss and succumbed to between day five and seven post-challenge. Thus, compared with the control mice, priming with NT60 virus can confer substantial level of cross-protection when the challenge dose of the heterosubtypic PR8 virus was low. However, severe body weight loss was observed in the group of NT60 virus-primed animals when high lethal dose of the PR8 virus was administered. One explanation for the results described above is that the robust cross-subtype protection conferred by priming with X31 virus is simply due to the ability of the virus to replicate more extensively in the lung of B6 mice compared to NT60 virus (the mean peak lung virus titers are approximately 10^7^ and 10^5^ EID_50_/lung, respectively), which may in turn stimulate a stronger memory immune response after priming. To rule out this possibility, we tested the ability of the X31 virus to induce cross-subtype protection against challenge with Taiwan virus (H1N1), where the NP_366_ epitope sequences between the two viruses differ by only one amino acid residue at the C-terminal bulge of the peptide-D^b^ ligand (Table 1). A different H3N2 priming virus, Memphis, was also used because it shares complete identity in NP_366_/D^b^ epitope sequence with Taiwan virus and replicates to a similar extent in B6 mouse lungs as the NT60 virus (mean peak lung virus titer: 10^5^ EID_50_/lung). As shown in Fig. 1, lower panel, priming with the Memphis virus conferred robust protection against either a low (3LD_50_) or high (9 LD_50_) lethal dose challenge of the heterosubtypic Taiwan virus. Note that the latter is the maximum lethal challenge dose achievable for this virus. In contrast, cross-protection against the Taiwan virus was significantly reduced in X31-primed mice.

Together, these results support previous observations demonstrating that priming with one subtype of live influenza A viruses can results in significant protection against subsequent infections with a different subtype of influenza A viruses [7]-[9]. Moreover, our results suggest that optimal protective heterosubtypic immunity conferred by CD8 T cells is only achieved when priming and challenge virus share the identical immunodominant CTL epitope(s). When the challenge viruses do not share the identical CTL epitopes with the priming viruses, the degree of heterosubtypic immunity against different subtypes of influenza A viruses appears to depend on the challenge dose. Complete cross-protection from death can be achieved, if the challenge dose is low, but protective heterosubtypic immunity is considerably reduced in the face of a high lethal challenge dose.

### Decreased heterosubtypic immunity is not primarily correlated to mucosal and serum antibodies, but to CD8 T cell subset

It has become increasingly clear that both T cell and B cell arms of the immune system may contribute to heterosubtypic immunity after influenza A virus infection, depending on the experimental systems used. In the present study, we used immune competent B6 mice in conjunction with intranasal priming and challenge to assess the capacity of the heterosubtypic immunity. It is possible that the differential capacity of the heterosubtypic immunity observed under this circumstance may be attributed to either the B cell or T cell arm of the immune system, or both.

**Figure 2:**
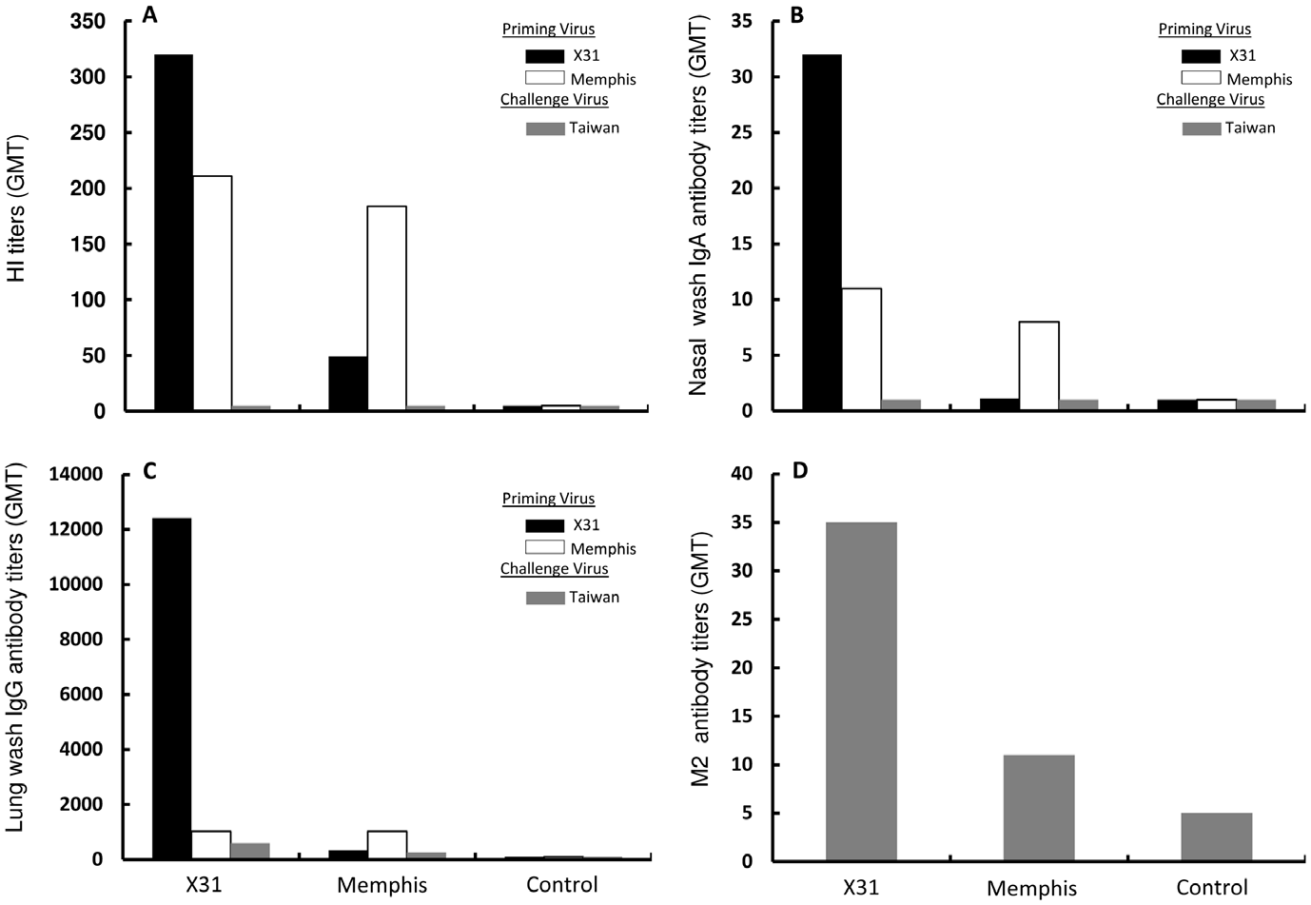
Strain-specific and cross-reactive antibodies following lethal challenge of primed mice with heterosubtypic influenza A virus. Five B6 mice per group were primed intranasally as described in figure 1. Serum, lung and nasal washes were sampled from individual animals at day 37 after priming and tested for serum HI titers (A), nasal wash IgA titers (B), lung wash IgG titers (C) against homotypic as well as heterosubtypic influenza A viruses as indicated. Serum IgG antibody titers to M2 protein was determined by an ELISA using synthetic M2e peptide as antigen (D).

To distinguish these possibilities, we first examined the possible correlation between serum HA-specific antibodies and heterosubtypic immunity. As expected, priming of B6 mice with either X31 or Memphis virus intranasally resulted in a robust serum antibody response to the homologous strains of the H3N2 viruses (Fig. 2A). Thirty-five days after the priming, serum HI geometric mean titers (GMT) to the homologous X31 and Memphis virus reached to 320 and 184, respectively. However, no subtype cross-reactive serum HI antibodies were detectable to the heterosubtypic Taiwan H1N1 virus following intranasal priming with either live H3N2 virus. Control mice did not show any detectable HI titers against any of the three influenza A viruses tested. These observations are consistent with the generally accepted understanding that serum HI and neutralizing antibodies to the HA glycoprotein of influenza A viruses are subtype-specific and their role in heterosubtypic immunity is minimal under normal circumstances.

We next examined whether IgA antibodies on the surface of the upper and lower respiratory tract of the primed mice contributed to heterosubtypic immunity following a lethal challenge. Fig. 2B shows that nasal washes obtained 35 days after priming with either X31 or Memphis virus contained IgA antibodies reactive to their homologous virus, although the GMT titers were low (32 and 8, respectively). However, cross-reactive nasal IgA antibodies to the heterosubtypic Taiwan H1N1 virus were not detected following intranasal priming with either H3N2 viruses. Similar results were obtained when lung IgG antibodies from the same animals were examined by a whole virus-ELISA, except that the amount of cross-subtype IgG antibodies to the Taiwan H1N1 virus were equally evident in the lung washes of both groups of the animals (Fig. 2C). Given that HA and NA from X31, e.g. A/Aichi/2/68, and A/Memphis/102/72 virus share high sequence identity (HA1: 96.6%, NA: 94.4%, respectively), it is not surprise that approximately equivalent amount of cross-subtype IgG antibodies to the Taiwan H1N1 virus were detected in the lung washes of both groups of the animals (Fig. 2C. GMT: 588 after priming with X31 versus 256 after priming with Memphis virus, respectively). Thus, neither nasal IgA nor lung IgG appeared to correlate with the differential capacity of the heterosubtypic immunity induced (Fig. 1).

Increasing evidence suggests that M2-specific antibodies induced by vaccination can provide cross-subtype protection against influenza A virus infections [4]. We monitored the levels of serum anti-M2 antibodies after priming with either X31 or Memphis virus using an M2e peptide-based ELISA. As shown in Fig. 2D, although M2-specific antibodies were detected, the GMT titers were low (35 after X31priming and only 5 after Memphis priming, respectively) and highly variable among individual mice. Therefore, we found no correlation between the levels of serum M2-specific antibodies induced by intranasal infection priming and the cross-subtype protection.

**Figure 3:**
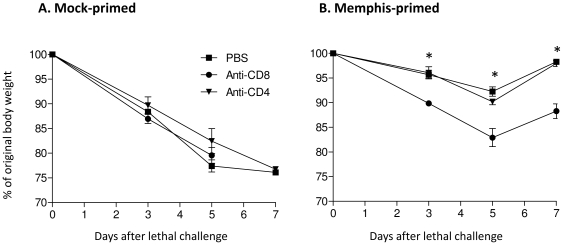
Effect of CD8- or CD4 T cell depletion on heterosubtypic immunity. Five B6 mice per group were primed intranasally as described in figure 1. Mock-primed (A) or Memphis virus-primed (B) animals were depleted of either CD8- or CD4 T cell subset *in vivo* as described in Materials and Methods. Monitoring of body weight loss after lethal challenge with Taiwan virus and expression of the results were described in detail in figure 1. One representative result is shown from two independent studies with similar results. Asterisk (*) indicates a statistically significant difference when CD8-depleted group was compared with PBS control group.

Next we examined the effect of T cell subset depletion on the capacity of heterosubtypic immunity described above. As shown in Fig. 3A, mock-primed animals experienced substantial body weight loss following lethal challenge with 9LD50 of Taiwan virus. All of the animals succumbed to infection by day 7 after lethal challenge, independent of CD8 or CD4 T cell depletion *in vivo*. In contrast, consistent with the results shown in Fig. 1, priming with Memphis virus led to strong resistance to subsequent lethal challenge with 9 LD_50_ of Taiwan virus (Fig. 3B). However, when the CD8 T cell subset was depleted *in vivo* from the Memphis-primed animals, significant weight loss was observed on day 5 after lethal challenge (mean percentage of original body weight: 92.3% versus 82.9% when PBS control was compared with CD8-depleted group, p = 0.0079). Depletion of CD4 T cell subset also resulted in slightly more severe weight loss compared to the PBS control group (90.2% versus 92.3%), but the difference was statistically not significant (P = 0.0952). Note that when compared to PBS-treated naïve animals, mice that was primed with Memphis virus and depleted of memory CD8 T cells showed significantly higher degree of cross-protection on day 5 after challenge with heterosubtypic Taiwan H1N1 virus (Mean body weight loss: 77.4% versus 82.9%, respectively. P = 0.0389). This is consistent with previous observation that primed CD4 T cell subset may also provide certain degree of cross-subtype protection under certain circumstances [2].

These results provide further evidence that multiple components of the immune system may be involved in the cross-subtype protection against heterosubtypic influenza A viruses, but CD8 T cell functions may be most closely correlated with heterosubtypic protection induced following intranasal inoculation with live influenza A virus.

### Decreased capacity of the heterosubtypic immunity is correlated with significantly reduced magnitude of the cross-reactive memory CD8 T cell response to the NP_366_/D^b^ variants

**Figure 4:**
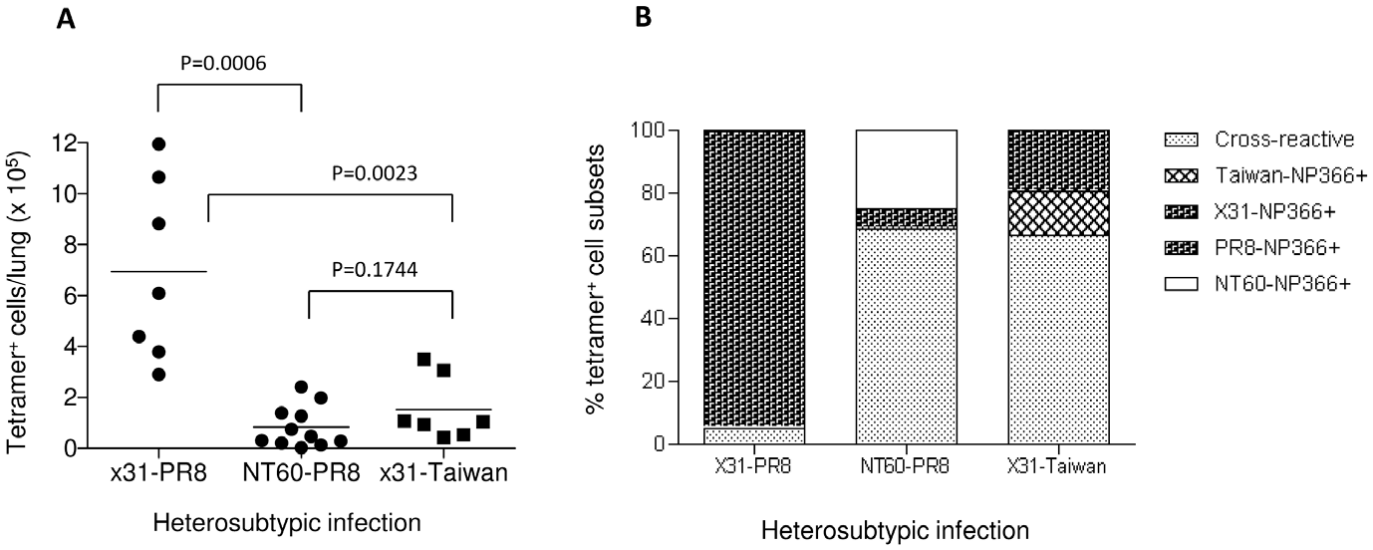
Magnitude and composition of specific and cross-reactive CD8 T cells to wild-type and variant NP_366_/D^b^ epitope after heterosubtypic influenza virus infections. Five to ten B6 mice per group were primed and challenged with lethal doses of heterosubtypic influenza A viruses as described in figure 1. Lung tissues were collected from individual animals five days after the lethal challenge and examined for the magnitude of the CD8 T cell response to the wild-type and variant NP_366_/D^b^ epitope (A). The mean percentages of the cell subsets from the same set of the data were used to assess the composition of the NP_366_/D^b^ response specific for the original NP_366_ priming sequence or cross-reactive to the NP_366_ challenge variants as indicated (B).

Early data obtained using ^51^Cr-release assay revealed that influenza virus-specific CD8 T cells are in general highly cross-reactive [30], [31]. However, the magnitude of cross-reactive CD8 T cell responses to influenza virus CTL epitope variants has not been quantitatively studied in the context of heterosubtypic immunity *in vivo*. We thus used a dual MHC class I tetramer technique to quantify the total numbers of the NP_366_/D^b^ -specific CD8 memory effector cells in the lung and the spleen of the mice following the sequential influenza A virus infection. As shown in Fig. S1A, flow cytometric analysis confirmed the specificity and cross-reactivity of these tetramers to homologous and heterologous NP_366_/D^b^ variants. As shown in Fig. 4A, priming with X31 virus followed by challenge with PR8 virus led to a massive expansion of the PR8-NP_366_/D^b^ -specific CD8 T cells in the lungs of the animals on day 5 post-challenge (6.94×10^5^ cell/lung). However, when NT60-primed mice were challenged with PR8 virus, the total number of tetramer-positive cells detected in the lungs was significantly lower (0.84×10^5^ cell/lung) compared with those obtained following X31-PR8 virus sequential infection (p = 0.0006). Similar results were obtained when X31-Taiwan virus sequential infection was performed. A detailed analysis of the compositions of the NP_366_/D^b^ tetramer-positive memory effector cell populations showed that following X31-PR8 virus sequential infection, over 95% of the responding memory CD8 T cells were directed to the NP_366_/D^b^ epitope shared by both virus strains (Fig. 4B and Fig S1B). CD8 T cells cross-reactive to the NT60-NP_366_/D^b^ variant were detectable, but at a very low frequency (<5%). In contrast, following either NT60-PR8 or X31-Taiwan sequential infection, the majority of the responding memory effector cells were cross-reactive to both the priming and challenge NP_366_/D^b^ epitope. In either case, only a small proportion of the cells were specific for the respective priming and the challenge NP_366_/D^b^ epitope. It is intriguing to note that the percentage of the CD8 T cells specific for the priming NT60-NP_366_/D^b^ epitope was considerably higher than those specific for the challenge PR8-NP_366_/D^b^ variant (24.5% versus 6.4%) following the NT60-PR8 sequential infection. However, such a biased memory CD8 T cell response was less pronounced following X31-Taiwan sequential infection (19.2% of X31-NP_366_/D^b+^CD8^+^ cells versus 14.2% of Taiwan-NP_366_/D^b+^CD8^+^ cells). These data clearly demonstrate that the quantity of the cross-reactive memory CD8 T cells is considerably diminished when priming and challenge viruses lack complete sequence identity of the NP_366_/D^b^ CTL epitope. Preliminary data indicate that the functional quality of the different subsets of the NP_366_/D^b^ memory effector cells may not differ considerably, as detection of intracellular IFNγ secretion following restimulation of the CD8 T cells with the homologous as well as the NP_366_ variant peptides did not reveal substantial differences for any population tested.(Fig. S2).

Together with the observations described above (Fig. 1 and 3), these results clearly indicate that the capacity of the heterosubtypic immunity mediated by the NP_366_/D^b^ CD8 T cells is closely associated with the sequence similarities between the priming and the subsequent challenge NP_366_/D^b^ epitopes. If both epitopes bear the identical NP_366_ sequence, strong heterosubtypic immunity can be anticipated, primarily owning to a robust expansion of the memory CD8 T cells triggered by the homologous challenge NP_366_/D^b^ epitope. When the challenge viruses do not share the identical sequence of the NP_366_/D^b^ epitopes with the priming viruses, two important factors may contribute to the reduced hetersubtypic immunity. First, considerably reduced expansion of the cross-reactive NP_366_/D^b^ cells triggered by the heterologous challenge NP_366_/D^b^ variants (Fig. 4). Another factor may be a biased memory CD8 T cell response toward the priming NP_366_/D^b^, but not the challenge NP_366_/D^b^ epitope, as was observed with the NT60-PR8 sequential infection (Fig. 4B). The reason for this phenomenon is not clear at present. It is possible that CTL original antigenic sin may exist under this circumstance. So far, this phenomenon has been only documented after infection with lymphocytic choriomeningitis viruses [32] or Dengue virus [33]. More comprehensive studies are needed to ascertain whether the phenomenon observed after NT60-PR8 sequential infection represents a generic CTL original antigenic sin phenomenon in the influenza A virus system, as such a phenomenon was not observed following a sequential delivery of X31-NP_366_ and Taiwan-NP_366_, which differ in one amino acid residue at position 7 of the NP_366_/D^b^ epitope.

### Limited plasticity of a dominant public TCR to the heterologous NP_366_/D^b^ variants may contribute to the decreased expansion of the cross-reactive memory CD8 T cells

**Figure 5:**
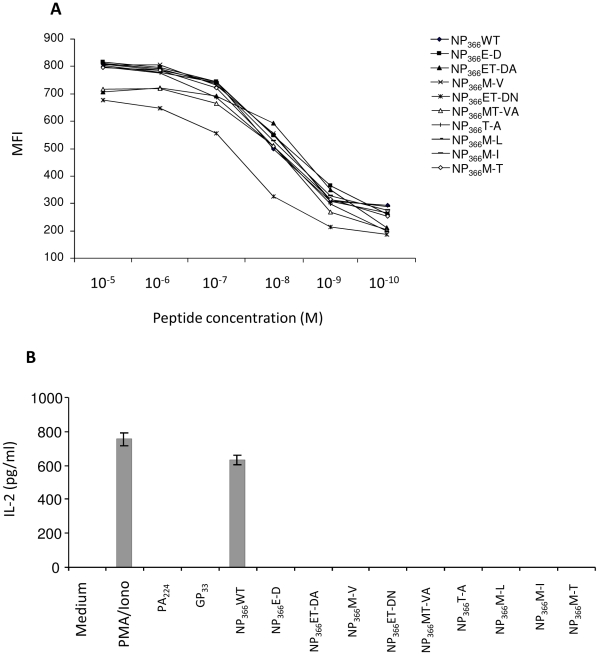
Plasticity of a dominant public TCR to NP_366_/D^b^ variants. Serially titrated amount of synthetic NP_366_/D^b^ peptides identified in table 1 were used to stabilize D^b^ expression on the surface of a TAP-deficient RMA-S cells in an RMA-S assay (A). The results were expressed as mean fluorescence intensity (MFI). The ability of the NP_366_/D^b^ variants to trigger activation of a T cell transfectant (clone A3-4) expressing the dominant public TCR specific for the PR8-NP_366_/D^b^ ligand, was assessed by an IL-2 assay (B). The data are representative of two independent experiments.

The data thus far suggest a limited TCR plasticity of the NP_366_/D^b^ -specific memory CD8 T cells for heterologous NP_366_/D^b^ variants. We and others have observed previously that public TCRs are strongly selected following a primary influenza A virus infection in B6 mice (up to 50%) [34], [35]. As a dominant public TCR that recognizes PR8-NP_366_/D^b^ epitope has been functionally expressed in the form of a stable transfectant [36], this allowed us to dissect to what extent the public TCR can cross-react to the naturally occurring NP_366_/D^b^ variants identified from the bank of NP sequences (Table 1). As shown in Fig. 5A, with the exception of the NP_366_ET-DN amino acid substitutions, all of the NP_366_ variants bound to the D^b^ molecule with similar high affinity. Surprisingly, when the ability of the public TCR to recognize these NP_366_ peptide variants was examined, only the cognate NP_366_/D^b^ ligand derived from the NP of the PR8 virus was able to trigger the activation of the TCR transfectant (Fig. 5B). Interaction between the nine NP_366_ peptide variants and the public TCR did not result in the production of IL-2 under the same experimental conditions. These results indicate that the high frequency of the public TCR in the memory CD8 T cell repertoire cannot tolerate any substitutions of the TCR contact residues within the original NP_366_ peptide sequence. Even a single conserved residue replacement at position 7 (D-E7) completely abolished the productive interaction between the TCR and the variant NP_366_/D^b^ ligands, as observed in the case of the Taiwan-NP_366_/D^b^ ligand (Table S1). It is conceivable that such a stringent requirement for sequence identity between the public TCR and its original NP_366_/D^b^ ligand may considerably reduce the cross-reactivity of memory CD8 T cells following re-infections with heterosubtypic influenza A viruses bearing NP_366_/D^b^ variants.

Taken together, our results reveal a significant limitation of an important immune effector responsible for heterosubtypic immunity to influenza A virus infection. We found that following primary infection, a high frequency of the NP_366_-specific memory T cells generated used the public TCRs that are strictly specific for the priming NP_366_/D^b^ epitope sequence. Upon re-exposure to heterosubtypic viruses that share the identical NP_366_ epitope sequence with the priming strain, robust memory CD8 response to the initial NP_366_/D^b^ ligand were obtained, leading to the generation of strong heterosubtypic immunity. However, if the challenge virus does not share the identical NP_366_ epitope sequence with the priming virus, expansion of the pre-existing, cross-reactive memory CD8 T cells may be limited, either due to their low frequency in the memory CD8 cell repertoire or possibly, a CTL original antigenic sin phenomenon under certain circumstances. Consequently, the heterosubtypic immunity generated is significantly reduced. This implies that efforts to promote CD8 T cell immunity-based vaccination strategies against heterosubtypic influenza A viruses may be most effective, only when the priming and the protective CTL epitopes targeted share the identical epitope sequences. Our findings re-emphasize that multiple immune components may be required for development of broad-protective influenza vaccines.

## Materials and Methods

### Ethics Statement

All animal research conducted in the present study was approved by CDC's Institutional Animal care and Use Committee (Approval number: 1619) and in an Association for Assessment and Accreditation of Laboratory Animal Care International-accredited facility.

### Influenza A viruses and infection

The influenza A viruses used in this study were the H3N2 viruses X31, a reassortant virus possessing the surface HA and NA glycoprotein of A/Aichi/2/68 (H3N2) and six internal genes of A/Puerto Rico/8/34 (H1N1; PR8), A/Northern Territory/60/68 (NT60), A/Memphis/102/72 (Memphis) and the H1N1 viruses PR8 and A/Taiwan/01/86 (Taiwan). All viruses were propagated in the allantoic cavity of 10-day old embryonated chicken eggs. All of the viral stocks were titered for HA units and 50% egg infectious dose (EID_50_). In addition, the 50% mouse infectious dose (MID_50_) and 50% lethal dose (LD_50_) for C57BL/6 (B6) mice were determined for the H3N2 and H1N1 viruses, respectively (Table S3).

Female B6 mice were purchased from Taconic (Albany, NY) and were used at 6-10 weeks of age. For priming, mice were inoculated intranasally with 250 MID_50_ of an H3N2 virus using Avertin as anesthesia. Between 35 and 45 days later, a time point where the memory T cells are generally considered to have established, the primed mice were challenged intranasally with lethal doses of H1N1 viruses as indicated. Mice that lost 25% of their original body weights were considered moribund and euthanized.

### Sera and tissue sampling

Immune sera from mice were collected from the orbital plexus on day 35–45 after priming. To collect lung and nasal washes, mice were sacrificed and the trachea was exposed. An 18-gauge cannula attached to a 1-ml syringe was inserted into the lungs through the incision in the trachea. The lungs were flushed repeatedly with a 1-ml volume of PBS buffer containing 1% bovine serum albumin. Nasal wash samples were recovered by flushing 1 ml of the PBS buffer through the tracheal incision forwarded into the nasal passage. The fluid expelled through the nares was collected in a petri dish and was flushed through the nose two more times. Lung and spleen tissues were sampled at the indicated time points and processed to give single cell suspensions for flow cytometry analysis as described previously [35].

### Synthetic peptides

NP_366_ peptides were synthesized either at New England Peptide, Inc., Fitchburg, MA, or at Division of Research Resources, CDC, Atlanta, GA. The purity of the synthesized peptides was >96%, as determined by HPLC analysis. All peptides had expected masses as confirmed by mass spectrometry.

### Influenza serology assays

Sera were treated with receptor-destroying enzyme from Vibrio cholerae (Denka-Seiken, Tokyo, Japan) before testing for the presence of H1 and H3-specific antibodies [37]. The hemagglutination-inhibition (HI) assay was performed using 4 hemagglutinating units of virus and 0.5% turkey red blood cells [37]. IgG and IgA antibodies were detected by an ELISA using sucrose gradient centrifugation purified viruses as antigens as described previously [38]. The ELISA end-point titers were expressed as the highest dilution that yielded an OD greater than the 2 times mean OD plus SD of similarly diluted negative control samples.

### *In vivo* depletion of CD8 and CD4 T cells

Mice were injected i.p. every third day either with 500 µg of purified rat anti-mouse CD8α mAb (clone 2.43), or 320 µg of purified rat anti-mouse CD4 mAb (clone GK1.5) or the same volume of PBS as control. Both mAb products were produced at Division of Research Resources, CDC, Atlanta, GA. The depletion started 3 days before viral infection and continued until the experiments were completed (day 10 after viral infection). The depletion started after priming and 3 days before secondary viral challenge with lethal dose of influenza A viruses and continued until the experiments were completed (day 10 after viral infection). Flow cytometric analysis confirmed that CD8 and CD4 T cells were undetectable in lung and spleen tissues during the entire 10-day period of observation, whereas the B cell subset was not affected by either of the depletion protocols.

### Flow cytometry

Influenza virus NP_366_ tetramers conjugated either with PE or APC were purchased from Beckman Coulter, Inc., (San Diego, CA). Immuno-staining of cells was performed as described previously [35] using the tetramers in combination with fluorochrome-conjugated anti-mouse CD3ε and CD3α mAb (BD Pharmingen). The results were expressed as either total numbers of tetramer^+^CD8^+^ cells per organ or the percentage of tetramer^+^CD8^+^ cells among total CD8 T cells.

### MHC-I-peptide binding

The binding affinity of the NP366 peptide variants to mouse H-2 D^b^ molecules was evaluated by measuring stabilization of MHC-I molecules on the surface of the TAP-deficient mutant cell line RMA-S according to a protocol described previously [26]. The results were expressed as mean fluorescence intensity (MFI) of RMA-S cells incubated with serially titrated amount of NP_366_ peptides.

### Determination of IL-2 production by the TCR transfectants

Generation of the A3-4 transfectant expressing a public TCR specific for PR8-derived NP_366_/D^b^ has been described in detail elsewhere [36]. A mouse Th1/Th2 cytokine Cytometric Bead Array (BD) was done as described previously to measure the levels of IL-2 in the culture supernatant following stimulation of the transfectant with the NP_366_ peptide variants in the presence of EL-4 cells as APCs. The detection sensitivity of the assay is 20 pg IL-2/ml.

### Statistical analysis

Data were analyzed with unpaired *t* test (Prism 5, GraphPad Software, Inc.) as indicated. P<0.05 is considered statistically significant.

## Supporting Information

Table S1 MHC class I-restricted immunodominant T cell epitopes of the influenza A viruses used in the present study.
(0.03 MB DOC)

Table S2 MHC class II-restricted immunodominant T cell epitopes of the influenza A viruses used in the present study.
(0.03 MB DOC)

Table S3 Infectivity of the virus stocks used in the present study.
(0.04 MB DOC)

Figure S1 NP366/Db-specific response after influenza A virus infection. B6 mice were infected intranasally with 250 MID50 of H3N2 influenza viruses as indicated. A. 10 days after primary infection, lung tissues were collected. Primary NP366/Db-specific CD8 response was examined by flow cytometry as described in detail in Material and Methods. CD3+CD8+ T cells were gated for analysis of NP366/Db+ cells. B. 40 days after primary infection, a second group of the animals were challenged intranasally with either 3 LD50 of PR8 virus or 3 LD50 of Taiwan virus. Lung and spleen tissues were collected on day 5 after lethal challenge. Memory NP366/Db-specific CD8 response was examined as described in A.
(0.44 MB TIF)

Figure S2 IFNγ secretion of NP366/Db-specific memory CD8 T cells. Single cells were prepared from the pooled spleen of B6 mice (5 mice per group) that were primed with x31 and subsequently challenged with lethal dose of either PR8 or Taiwan virus as described in Fig. S1. The cells were then stimulated with either 0.5 mg/ml of the NP366 peptides as indicated or no peptide in the presence of IL-2. Amino acid sequences of the NP366 peptides used are shown in table S1. After 16 hour in culture, the cells were stained with anti-mouse IFNγmAb intracellularly followed by surface staining with anti-CD3 and CD8 mAbs. CD3+CD8+ T cells were gated for flow cytometric analysis. The data are representative of two independent experiments.
(0.24 MB TIF)
