# Supplementary material for: Significant Impact of Sequence Variations in the Nucleoprotein on CD8 T Cell-Mediated Cross-Protection against Influenza A Virus Infections
Source: PLoS One. 2010 May 11;5(5):e10583. doi: 10.1371/journal.pone.0010583 (PMC2868023; doi:10.1371/journal.pone.0010583)
Supplement: Table S1 — MHC class I-restricted immunodominant T cell epitopes of the influenza A viruses used in the present study. (0.03 MB DOC) [file pone.0010583.s001.doc]

**Table S1. MHC class I-restricted immunodominant T cell epitopes of the influenza A viruses used in the present study^1^**

| Virus strain | Subtype | NP_366_/D^b^ | PA_224_/D^b^ | PB1_703_/K^b^ |
| --- | --- | --- | --- | --- |
| A/NT/60/68 | H3N2 | A S N E N M D A M | S S L E N F R A Y V | S S Y R R P V G I |
| X31 | H3N2 | A S N E N M E T M | S S L E N F R A Y V | S S Y R R P V G I |
| A/Memphis/102/72 | H3N2 | A S N E N M D T M | S C L E N F R A Y V | S S Y R R P V G I |
| A/Puerto Rico/8/34 | H1N1 | A S N E N M E T M | S S L E N F R A Y V | S S Y R R P V G I |
| A/Taiwan/01/86 | H1N1 | A S N E N M D T M | S C L E N F R A Y V | S S Y R R P V G I |

^1^Whole genome sequencing was performed for all of the influenza A viruses listed above at Influenza Sequencing Core, Influenza Division, CDC. No mutations were identified in the regions of the viral proteins corresponding to the immunodominant MHC class I and class II T cell epitopes in the stock viruses used for this study compared with amino acid sequences retrieved from the NCBI database.
