# Supplementary material for: Significant Impact of Sequence Variations in the Nucleoprotein on CD8 T Cell-Mediated Cross-Protection against Influenza A Virus Infections
Source: PLoS One. 2010 May 11;5(5):e10583. doi: 10.1371/journal.pone.0010583 (PMC2868023; doi:10.1371/journal.pone.0010583)
Supplement: Table S2 — MHC class II-restricted immunodominant T cell epitopes of the influenza A viruses used in the present study. (0.03 MB DOC) [file pone.0010583.s002.doc]

**Table S2. MHC class II-restricted immunodominant T cell epitopes of the influenza A viruses used in the present study**

| Virus strain | Subtype | HA_211_/I-A^b^ | NP_311_/I-A^b^ |
| --- | --- | --- | --- |
| A/NT/60/68 | H3N2 | Y V Q A S G R V T V S T R R S | Q V Y S L I R P N E N P A H K |
| A/x31 | H3N2 | Y V Q A S G R V T V S T R R S | Q V Y S L I R P N E N P A H K |
| A/Memphis/102/72 | H3N2 | Y V Q A S G R V T V S T R R S | Q V Y S L I R P N E N P A H K |
| A/Puerto Rico/8/34 | H1N1 | y q n e n a y v s v v t s n y | Q V Y S L I R P N E N P A H K |
| A/Taiwan/01/86 | H1N1 | Y H T e n a y v s v v S s H y | Q V Y S L I R P N E N P A H K |
