# Supplementary material for: Significant Impact of Sequence Variations in the Nucleoprotein on CD8 T Cell-Mediated Cross-Protection against Influenza A Virus Infections
Source: PLoS One. 2010 May 11;5(5):e10583. doi: 10.1371/journal.pone.0010583 (PMC2868023; doi:10.1371/journal.pone.0010583)
Supplement: Table S3 — Infectivity of the virus stocks used in the present study. (0.04 MB DOC) [file pone.0010583.s003.doc]

**Table S3. Infectivity** of the virus stocks used in the present study

| Strain | Subtype | HAU^1^ | EID_50_^2^ | MID_50_^3^ | LD_50_^3^ |
| --- | --- | --- | --- | --- | --- |
| X31 | H3N2 | 1:1024 | 10^8.75^ | 10^2.25^ | n.d. |
| A/Memphis/102/1972 | H3N2 | 1:1024 | 10^9.5^ | 10^2.50^ | n.d. |
| A/NT/60/1968 | H3N2 | 1:1024 | 10^8.75^ | 10^2.25^ | n.d. |
| A/Taiwan/01/1986 | H1N1 | 1:512 | 10^8.25^ | 10^1.75^ | 4 x 10^5^ |
| A/Puerto Rico/8/34 | H1N1 | 1:1024 | 10^9.5^ | 10^1.5^ | 1x 10^4^ |

^1^ Hemagglutination Unit (HAU) was expressed as the highest dilution of a virus stock to cause hemagglutination of Turkey red blood cells in a 50 μl of reaction volume.

^2^50% Egg Infectious Dose (EID_50_) was defined as the highest dilution of a virus stock to infect 50 percent of 10-day old embryonated chicken egges.

^3^50% Mouse Infectious Dose (MID_50_) and 50% Lethal dose (LD_50_) were determined in C57BL/6 mice and expressed as EID_50_ required to give 1 MID_50_ and 1 LD_50_, respectively. n.d.: not determined.
