# Supplementary figures and images for: Significant Impact of Sequence Variations in the Nucleoprotein on CD8 T Cell-Mediated Cross-Protection against Influenza A Virus Infections
Source: PLoS One. 2010 May 11;5(5):e10583. doi: 10.1371/journal.pone.0010583 (PMC2868023; doi:10.1371/journal.pone.0010583)

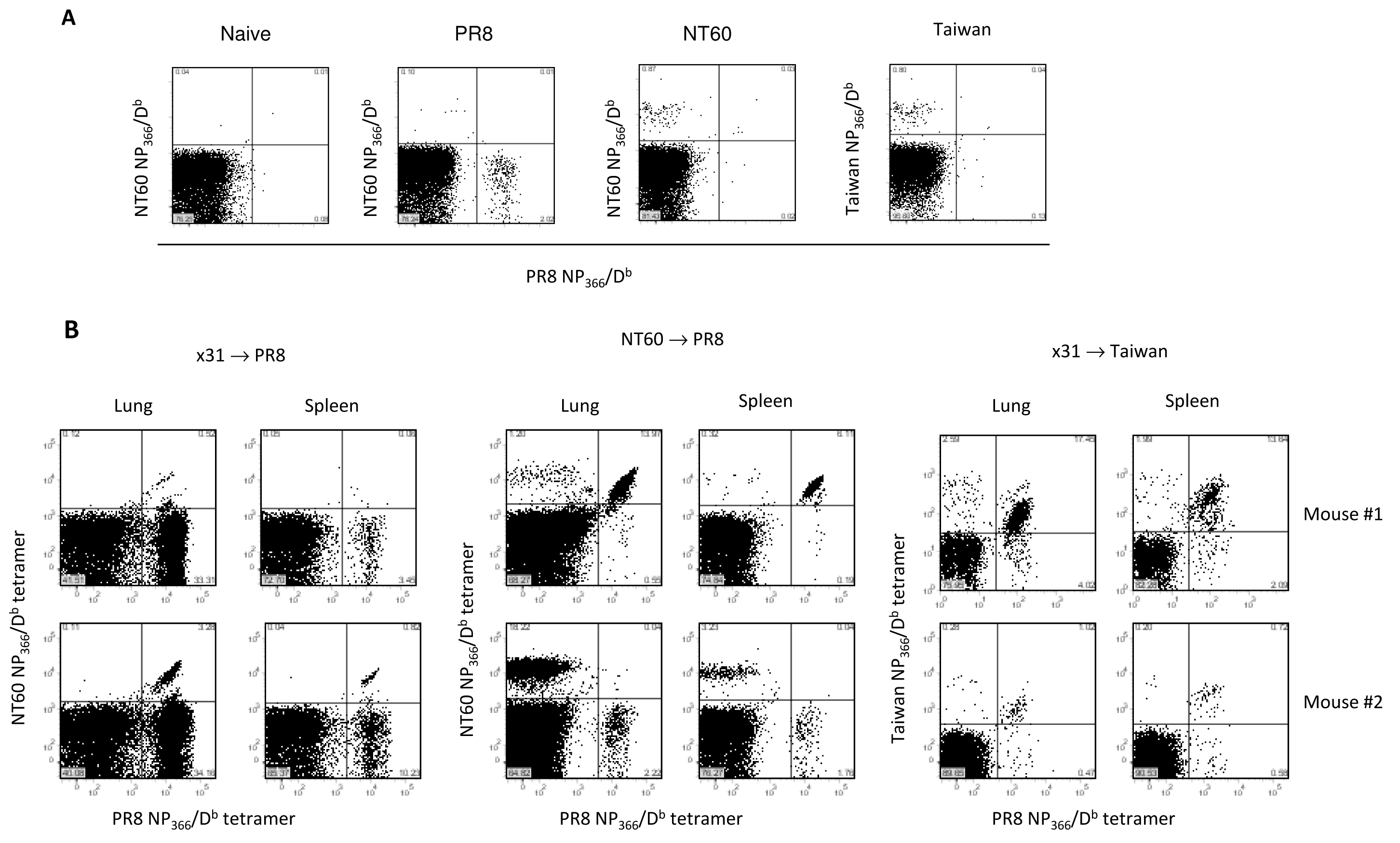

Supplement: Figure S1 — NP366/Db-specific response after influenza A virus infection. B6 mice were infected intranasally with 250 MID50 of H3N2 influenza viruses as indicated. A. 10 days after primary infection, lung tissues were collected. Primary NP366/Db-specific CD8 response was examined by flow cytometry as described in detail in Material and Methods. CD3+CD8+ T cells were gated for analysis of NP366/Db+ cells. B. 40 days after primary infection, a second group of the animals were challenged intranasally with either 3 LD50 of PR8 virus or 3 LD50 of Taiwan virus. Lung and spleen tissues were collected on day 5 after lethal challenge. Memory NP366/Db-specific CD8 response was examined as described in A. (0.44 MB TIF) [file pone.0010583.s004.tif]

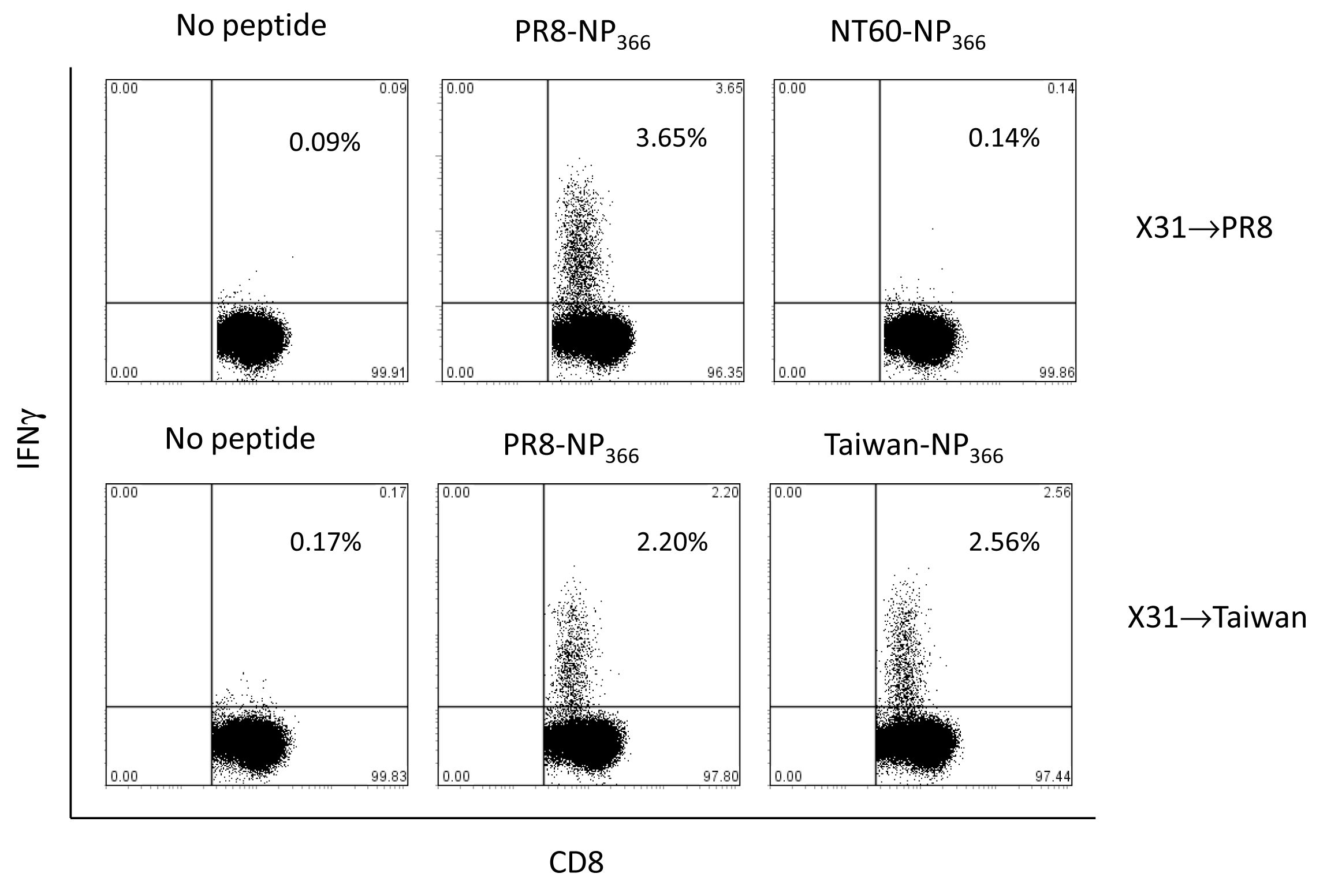

Supplement: Figure S2 — IFNγ secretion of NP366/Db-specific memory CD8 T cells. Single cells were prepared from the pooled spleen of B6 mice (5 mice per group) that were primed with x31 and subsequently challenged with lethal dose of either PR8 or Taiwan virus as described in Fig. S1. The cells were then stimulated with either 0.5 mg/ml of the NP366 peptides as indicated or no peptide in the presence of IL-2. Amino acid sequences of the NP366 peptides used are shown in table S1. After 16 hour in culture, the cells were stained with anti-mouse IFNγmAb intracellularly followed by surface staining with anti-CD3 and CD8 mAbs. CD3+CD8+ T cells were gated for flow cytometric analysis. The data are representative of two independent experiments. (0.24 MB TIF) [file pone.0010583.s005.tif]
